# Feedback Regulation of Kinase Signaling Pathways by AREs and GREs

**DOI:** 10.3390/cells5010004

**Published:** 2016-01-25

**Authors:** Irina Vlasova-St. Louis, Paul R. Bohjanen

**Affiliations:** 1 Department of Medicine, University of Minnesota, Minneapolis, MN 55455, USA; bohja001@umn.edu; 2 Center for Infectious Diseases and Microbiology Translational Research, University of Minnesota, Minneapolis, MN 55455, USA; 3 Department of Microbiology, University of Minnesota, Minneapolis, MN 55455, USA

**Keywords:** posttranscriptional gene regulation, ARE- GRE- mRNA stability, CELF1, ELAVL1, ZFP36, kinase signaling

## Abstract

In response to environmental signals, kinases phosphorylate numerous proteins, including RNA-binding proteins such as the AU-rich element (ARE) binding proteins, and the GU-rich element (GRE) binding proteins. Posttranslational modifications of these proteins lead to a significant changes in the abundance of target mRNAs, and affect gene expression during cellular activation, proliferation, and stress responses. In this review, we summarize the effect of phosphorylation on the function of ARE-binding proteins ZFP36 and ELAVL1 and the GRE-binding protein CELF1. The networks of target mRNAs that these proteins bind and regulate include transcripts encoding kinases and kinase signaling pathways (KSP) components. Thus, kinase signaling pathways are involved in feedback regulation, whereby kinases regulate RNA-binding proteins that subsequently regulate mRNA stability of ARE- or GRE-containing transcripts that encode components of KSP.

## 1. Introduction 

Protein kinases are central components of intracellular networks that regulate a vast number of cellular processes [1]. Kinase-mediated signals are important regulators of pathways that control cell fate decisions during development and stress responses. Kinases are components of signal transduction pathways that regulate responses to extracellular or intracellular signals [2,3,4]. Kinases mediate post-translational effects on gene expression: through direct phosphorylation of target proteins, or through indirect effects such as the phosphorylation and activation of other kinases or phosphatases involved in the same signaling pathway [5]. 

In addition to the role of transcriptional regulatory mechanisms in the regulation of kinase gene expression, kinases and other kinase signaling pathway (KSP) components are regulated at the posttranscriptional level through mRNA degradation or stabilization. The stability of KSP mRNAs can be regulated in the cytoplasm where kinase expression is often induced by different stimuli through transcript stabilization and increased translation. KSP mRNAs can also be rapidly degraded by RNA-binding proteins (RNA-BPs) that bind mRNAs through specific motifs, usually located in the 3’ untranslated regions (3’UTRs) [6,7]. AU-rich elements (AREs) and GU-rich elements (GREs), discussed below, are specific motifs that serve as binding sites for RNA-BPs that regulate mRNA stability/translation. Binding to target transcripts by ARE-binding proteins, such as ZFP36, or GRE-binding proteins, such as CELF1, usually leads to rapid mRNA degradation and down-regulated gene expression. In contrast, binding by ELAVL1 to ARE or GRE-containing target transcripts usually leads to transcript stabilization. As discussed below, each of these RNA-BPs is a substrate for phosphorylation by cellular kinase pathways, and phosphorylation of these RNA-BPs significantly regulates their mRNA-(de)stabilizing function. 

The level of an mRNA within an individual cell depends on both its rate of synthesis and the rate of decay [8]. The recognition of mRNAs by specific RNA-BPs (as well as miRNAs and lncRNAs) is mediated through sequence or structural elements in the target mRNA [9,10]. Target sequences of RNA-BPs that regulate mRNA degradation are often found in the 3’UTRs of those transcripts, and binding by regulatory RNA-BPs, determines the degradation or stabilization of bound transcripts [11]. Many RNA-BPs are regulated through phosphorylation by upstream kinase signaling cascades. Studies from independent laboratories have led to the identification of numerous kinases, such as MEKK, PI3K, AKT, PKC, MAPK/p38, CHK, JNK, that use RNA-BPs as substrates [12,13,14,15]. There is no unified common effect of kinases on the function of different RNA-BPs. Phosphorylation of an RNA-BP can decrease or increase binding affinity for RNA, alter its ability to regulate translation, or direct its relocalization to different intracellular compartments. These effects depend upon which amino acid position of the RNA-BP is phosphorylated or if a RNA-BP is mono-phosphorylated or extensively phosphorylated at multiple positions. It is evident, however, that signals transmitted through kinase cascades lead to the phosphorylation of RNA-BPs, thereby regulating their function outcome in posttranscriptional gene expression [16,17,18,19]. 

In this article, we review our understanding of the mechanisms by which kinases control the function of RNA-BPs that bind to AREs or GREs, highlighting studies regarding ZFP36, ELAVL1 and CELF1. These proteins influence many aspects of mRNA metabolism, including regulation of the degradation of networks of target transcripts that encode multiple KSP components. Not only are these RNA-BPs regulated thorough phosphorylation by kinases, these RNA-BPs function as feedback regulators of kinase expression by controlling stability of transcripts encoding KSP components. We describe how phosphorylation affects binding by ZFP36, ELAVL1 and CELF1 to target transcripts and thereby changes the expression of networks of target transcripts that encode components of KSPs that control important cellular processes including immune activation, proliferation, and apoptosis. 

## 2. AREs and GREs

It was noted over a quarter of a century ago that mRNAs exhibit substantial variations in turnover rates upon exposure to different cell stimuli [20]. More recently, genome-wide analyses of mRNA transcript half-lives showed that many labile transcripts contain conserved sequence elements in their 3’UTRs [21]. Two large networks of mRNAs contain either conserved AU-rich elements (AREs) or GU-rich elements (GREs) in their 3’UTRs and are regulated through mRNA stability or translation in mammalian cells [22]. In general, transcripts containing functional AREs or GREs have short half-lives, although they can rapidly stabilized in different cell types or stimulation conditions through complex posttranscriptional mechanisms. Bioinformatics searches throughout the human transcriptome have provided computational estimation of sequence characteristics and nucleotide lengths for ARE and GRE motifs, for mRNA to be unstable. The classification of AREs and GREs has been described in multiple manuscripts [23,24,25,26], and an overview is shown in Table 1.

ARE or GRE-containing transcripts in clusters I and II contain 4 or more overlapping AUUUA or GUUUG pentamers and are each represented by only a few hundred transcripts. Most of the transcripts in these clusters are cytokines, transcription factors and early response genes. Clusters III through V contain shorter sequences with less sequence repetition, and these clusters contain up to several thousand members. Several functional classes of mRNAs contain clusters III-V, including transcripts encoding proteins involved in cell signaling, cell to cell communication, cell cycle and apoptosis. With gradually increasing overall U-richness in these clusters, the destabilizing potency decreases [27]. Nevertheless, numerous studies demonstrated that mutation in these conserved cis-acting elements resulted in changes in mRNA stability and binding preferences for RNA-BPs (reviewed in Reference [28]). Numerous proteins interact with AREs (ELAVL1, ZFP36, KSRP, HNRNPD, TIA1, TIAR, and others) and GREs (CELF1, CELF2, ELAVL1, TDP43), but there is considerable overlap in the binding sites of ARE- and GRE-binding proteins that warrants further investigation. For example, there was significant overlap noticed between the sets of transcripts bound by ELAVL1 and CELF1 in different cell types [29,30,31,32]. The next sections describe what is known about the impact of phosphorylation on the functions of the ZFP36, ELAVL1 and CELF1 proteins in mRNA stability regulation.

**Table 1:** Structural and functional classifications of AU-rich elements (AREs) and GU-rich elements (GREs).

ARE Sequences	Functional Categories	Cluster	GRE Sequences	Functional Categories
AUUUAUUUAUUUAUUUAUUUA	Cytokines; Growth factors; Cell signaling; Apoptosis	**I**	GUUUGUUUGUUUGUUUGUUUG	Transcription factors; Cell cycle; Cell metabolism; Cell-cell communication
AUUUAUUUAUUUAUUUA		**II**	GUUUGUUUGUUUGUUUG	
WAUUUAUUUAUUUAW		**III**	GUKUGUUUGUKUG	
WWAUUUAUUUAWW		**IV**	KKGUUUGUUUGKK	
WWWWAUUUAWWWW		**V**	KKKU/GUKUG/UKKK	

The ARE and GRE mRNAs were clustered (with allowance for one mismatch) into five sub-classes based on the number of pentameric repeats (AUUUA or GUUUG) and surrounding sequences. W indicates A or U. K indicates G or U. This table was built based on previous publications [23,24,25,26].

## 3. Regulation of ZFP36 Function by Phosphorylation

ARE-binding proteins regulate the expression of a large network of genes that control a variety of cellular processes including immune activation, cytokine expression, and stress responses [33]. Over the last three decades, numerous ARE-binding proteins were identified in a variety of cell types, and many ARE-binding proteins are involved in regulation of transcript half-life [34,35,36]. The best-characterized ARE-binding protein that functions to destabilize mRNA is ZFP36 (also called tristetraprolin or TTP) [37,38,39]. ZFP36 is a member of a family of zinc finger RNA-binding proteins that binds to ARE sequences and regulates mRNA decay [40,41]. ZFP36 promotes mRNA decay by recruiting components of the mRNA degradation machinery to ARE-containing transcripts, including nucleases, deadenylases, and exosome components [42,43,44,45]. The ability of ZFP36 to bind to and recruit components of the cellular RNA decay machinery to ARE-containing transcripts is regulated by phosphorylation following immune cell activation. For example, ZFP36-mediated decay is regulated by phosphorylation of ZFP36 by p38 MAPK-activated protein kinase 2 (MK2) following LPS-stimulation of macrophages [46]. Phosphorylation of ZFP36 prevents ZFP36 from recruiting deadenylases [47] to the bound transcript by promoting ZFP36’s association with 14-3-3 proteins, while maintaining ZFP36’s ability to bind to the ARE [48]. Through this mechanism, ARE-containing ZFP36 target transcripts are stabilized and expressed at higher levels, allowing for effective immune responses [49]. Eventually ZFP36 phosphorylation is reversed by the phosphatase PP2A, allowing ZFP36 to return to its baseline function to mediate the rapid decay of ARE-containing transcripts [50]. Dysregulation of this balance between kinase and phosphatase activity on ZFP36 function can lead to abnormal target transcript expression and ultimately result in disease states such as autoimmunity or cancer [51]. Recently developed mouse strains that express unphosphorylated S52 and S178 ZFP36 exhibit weak immune responses [52]. Thus, transient phosphorylation of ZFP36 during an immune response promotes stabilization of ARE-containing transcripts, and subsequent dephosphorylation of ZFP36 allows these transcripts to be degraded [53,54].

## 4. Regulation of CELF1 Function by Phosphorylation

**Figure 1:**
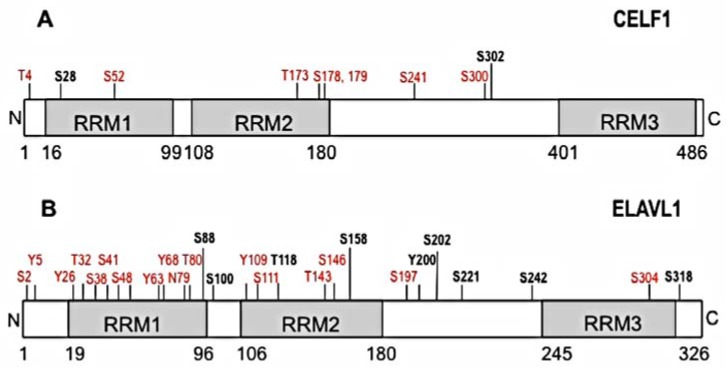
Schematic representation of predicted and verified phosphorylation sites on CELF1 and ELAVL1. Confirmed phosphorylation sites are depicted in bold black and predicted phosphorylation sites are depicted in red. The figure was built based on available information in PhosphoSitePlus software, Cell Signaling Technology, Inc. and publications cited in the text. (**A**) Phosphorylation sites for CELF1 (S28, S302) are implicated in changes in RNA binding affinity and altered mRNA splicing, translation and decay; (**B**) Phosphorylated residues for ELAVL1 are implicated in changes in intracellular localization (T118, S158, Y200, S202, S221, S242, S318), and mRNA translation (S158, S221).

CELF1 is an RNA-BP that regulates a network of transcripts that control important cellular functions such as cell growth and apoptosis by binding to GREs in the 3’UTR of target transcripts [55]. Along with ELAVL1, discussed below, CELF1 is a prototypic member of a family of RNA-binding proteins known as the CUGBP1 and ELAV-like family (CELF) of proteins (Figure 1) [27]. Each member of the family of CELF proteins contains three RNA recognition motifs (RRMs) that bind to RNA [56,57,58,59]. Binding sites for CELF proteins are usually U-rich, but binding sites may contain other nucleotides such as A or G. In addition to the canonical GRE, CELF1 binds to U-rich, GU-rich sequences, or GU-repeat sequences within pre-mRNA introns or mRNA 3’UTRs [60,61,62,63]. 

CELF1 is a known phosphoprotein with multiple predicted phosphorylation sites (Figure 1A). CELF1 phosphorylation appears to regulate several functions such alternative splicing, translation, and mRNA decay [64,65,66,67]. Activation of the cyclin D3-Cdk4/6 signaling cascade leads to phosphorylation of CELF1 at S302, affecting binding of CELF1 to eIF2α and influencing the rates of translation of several mRNAs (e.g., C/EBPbeta, CDKN1A) [68]. Phosphorylation of CELF1 by eIF2α stress kinases (e.g., PKR and PERK) facilitates binding of CELF1 to eIF2α and TIA1, altering the binding by CELF1 to pro-survival mRNA targets and trigger translational inhibition [69]. CELF1 phosphorylation by AKT kinase pathway at S28 in normal muscle myoblasts regulates the translation of CELF1 target transcripts during myocyte differentiation and murine heart development [70]. In addition, PKCα/β and downstream kinase-dependent phosphorylation of CELF1 at serine 28 (and possibly S52, 178, 179, 241, 300, 302) are involved in proper murine heart development [71]. Overall, phosphorylation of CELF1 regulates its function in various systems by causing changes in RNA binding which influences CELF1-regulated processes such as splicing, translation or mRNA decay.

CELF1 phosphorylation seems to be important in regulating immune responses. T cell activation through the T cell receptor and CD28 co-receptor leads to phosphorylation of CELF1, followed by inhibition of mRNA binding and stabilization of numerous GRE-containing target transcripts. In this case, the site of phosphorylation is not known, but this type of T cell activation is driven by PKCθ and other kinases, including MAP kinases and PI3 kinases (reviewed in Reference [72]). Possibly, these kinases are induced through T cell receptor and co-receptor stimulation, leading to transient phosphorylation of CELF1. The resulting loss of binding by CELF1 to GRE-containing target transcripts leads to the transient stabilization and up-regulation of these transcripts required for cellular proliferation. Subsequent dephosphorylation of CELF1 by an unknown phosphatase allows CELF1 to regain its RNA-binding and mRNA decay functions as the T cells return to a quiescent state. 

Through microarray-based mRNA decay profiling and identification of CELF1 target transcripts, we identified a number of GRE-containing transcripts that were CELF1 targets in normal but not malignant T cells even though they were expressed in both types of cells. We found CELF1 phosphorylation at serine 28 in malignant T cell lines, but not in resting or activated normal T cells, suggesting that a kinase capable of phosphorylating CELF1 at this site is active in malignant but not in normal T cells. In addition to phosphorylation at S28 in malignant T cells, we identified hyperphosphorylation of CELF1 at multiple unmapped sites. Overall, phosphorylation of CELF1 at S28 or other sites may lead to the altered CELF1 function that we observed in malignant T cells [63]. 

Hyperphosphorylation of CELF1 has been reported as a major contributor to pathogenesis in myotonic dystrophies, such as myotonic dystrophy type 1 (DM1) [73]. In DM1, PKC is activated by the accumulation of long CUG-repeats within DMPK mRNA, producing a toxic RNA effect [66]. In DM1 patient cells and mouse models of the disease, abnormal splicing patterns occur due to the accumulation of the stabilized CELF1 protein in the nucleus [74]. In transgenic mouse models of DM1, mice treated with specific inhibitors of the PKC pathway showed amelioration of cardiac abnormalities associated with the disease phenotype, presumably by limiting CELF1 phosphorylation [75]. PKCα/βII was recently discovered to be involved in CELF1 hyperphosphorylation in a mouse model of diabetes, also causing alternative splicing abnormalities in diabetic hearts [71]. More work is needed to understand how phosphorylation of CELF1 influences its function in normal and disease states.

Overall, a sizable body of evidence shows that phosphorylation affects many functions of CELF1 in posttranscriptional gene regulation. The effects of different kinase signaling may even cause a switch in cytoplasmic CELF1 functions (e.g., from mRNA destabilization to mRNA translation), or causes a switch in CELF1 localization from cytoplasmic to nuclear, since different functions of CELF1 are carried out in different compartments [76]. Phosphorylated CELF1 could potentially be sequestered to p–bodies or stress granules [77], which would obviously impair its ability to perform its cytoplasmic translation or mRNA decay functions [78]. Dramatic changes in substrate preference following phosphorylation might explain why unmodified recombinant CELF1 prefers only GU-rich sequences while some studies report binding to CUG- and GC-rich sequences in cell extracts [60,79,80]. The exact kinase pathways that regulate the activity and functional diversity of CELF1 remain unknown. Characterization of the precise sites of CELF1 phosphorylation and identifying the protein binding partners of CELF1 are needed to shed light on the multiple effects of CELF1 on RNA. As discussed below, CELF1 target transcripts include mRNA transcripts that encode kinases and KSP components that may regulate phosphorylation of CELF1. 

## 5. Regulation of ELAVL1 Function by Phosphorylation

ELAVL1 is another member of the CELF family of RNA-binding proteins whose function has been extensively studied in cellular models. ELAVL1 can regulate alternative splicing, processing, stabilization or mRNA translation (reviewed in Reference [81]). ELAVL1 is often considered an ARE-binding protein, because it binds to and mediates the stabilization of ARE-containing transcripts [34]. In addition to ARE sequences, ELAVL1 also binds to a variety of U-rich and GU-rich sequences, and there is considerable overlap between targets of CELF1 and ELAVL1 [27,35]. Like other CELF family members, ELAVL1 has three RRMs (Figure 1B), and each can interact with RNA individually, or through oligodimerization [42,82]. As described below, the phosphorylation of ELAVL1 protein may enhance or diminish its function as a transcript stabilizer by modifying its affinity for binding to RNA. 

In stressed cells, phosphorylation of ELAVL1 appears to represent a protective mechanism to help stressed cells survive, making this protein, and upstream kinases, attractive pharmacological targets [83,84]. ELAVL1 is rapidly phosphorylated following exposure of cells to different stress stimuli, such as lipopolysaccharide, growth factors, ultraviolet radiation, oxidative stress, DNA damage, heat or mechanical stresses (e.g., shear stress) [85,86,87,88,89]. Although in many cases, the phosphorylation site(s) are not known, it appears that ELAVL1 phosphorylation regulates its function. For example, during oxidative stress (exposure to hydrogen peroxide) CHK2 phosphorylates ELAVL1 at S88, S100 and T118 (within RRM1 and RRM2, see Figure 1B). In this case, ELAVL1 phosphorylation interrupts binding and prevents stabilization of ARE-containing mRNA [90]*.*

ELAVL1 phosphorylation also changes its ability to shuttle between the nucleus and cytoplasm. During the normal cell cycle, ELAVL1 is phosphorylated by CDK1 or CDK5 at S202 [91,92,93]. Phosphorylation by CDK1 kinase causes the retention of ELAVL1 in the nucleus during the G2/M cell cycle stage [91]. At this stage of the cell cycle, ELAVL1 phosphorylation correlates with increased degradation of ARE-containing transcripts. 

Another example where ELAVL1 phosphorylation regulates its cytoplasmic localization and increased ARE-binding activity is the phosphorylation by PKC isozymes [94,95,96]. Phosphorylation of ELAVL1 at S158, S221 and S242, within its RRM2 and nucleocytoplasmic shuttling region, leads to increased ELAVL1 localization and activity in the cytoplasm [97,98]. In most systems studied, cytoplasmic ELAVL1 appears to mediate the stabilization and increased translation of bound mRNA [99,100,101], but the effect of phosphorylation on the function of ELAVL1 seems to depend on the cellular system and site of phosphorylation. 

## 6. Regulation of Expression of KSP Components by RNA-BPs

**Figure 2:**
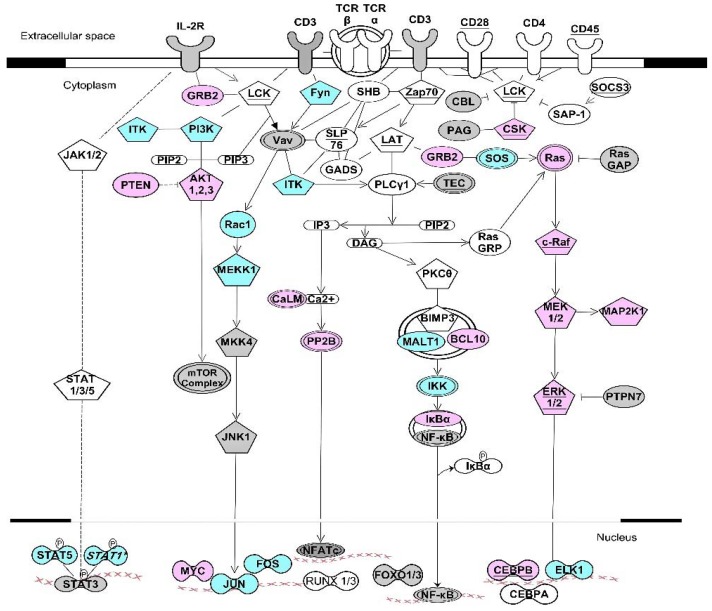
Kinase signaling pathways involved in T cell receptor activation. The network diagram depicts coordinate regulation of ARE- and GRE-containing transcripts involved in T cell activation. Colored transcripts (blue, pink, or grey) were transiently upregulated after T cell activation followed by destabilization and downregulation [103]. Pentagon-shaped nodes represent kinase transcripts. Transcripts marked with underlined text are CELF1 targets in resting T cells [61]. Transcripts in blue are targets of both CELF1 and ELAVL1 [31,61]. Transcripts in pink are targets of both ELAVL1 and ZFP36 [31,104]. Transcripts in grey are targets of ELAVL1 [31] but not CELF1 or ZFP36. Underlined pink nodes represent transcripts that are targets of all three proteins. Arrows indicate direct interactions and/or activations. Blunt-ended lines indicate inhibitory effects. This network diagram was built using Ingenuity Pathway Assistant Software.

Numerous transcripts encoding kinase signaling pathways, including kinases and phosphatases, are targets of RNA-BPs, suggesting that feedback regulation of KSP components occurs through regulated mRNA stability. It is becoming clear that KSP components are regulated at posttranscriptional levels by RNA-BPs that bind to AREs or GREs. For example, we have observed this type of feedback inhibition following activation of primary human T cells [102]. Numerous ARE- or GRE-containing transcripts that encode KSP components display transiently increased expression shortly after T cell activation, followed by a decrease in their expression later in the activation process (Figure 2). 

The increased expression of these transcripts appears to be due to increased transcription as well transcript stabilization as a result of inactivation of destabilizing RNA-BPs such as ZFP36 and CELF1 through transient phosphorylation [13,61]. Subsequently, ZFP36 and CELF1 become dephosphorylated and regain their mRNA decay function, leading to decreased expression of transcripts encoding KSP components. Figure 2 shows a network of short-lived ARE- and GRE-containing transcripts that encode KSP components involved in T cell signaling that are transiently induced following T cell activation and subsequently exhibit decreased expression [103]. Many of these ARE- or GRE-containing transcripts have been shown to be targets of RNA-BPs such as ZFP36, CELF1, and/or ELAVL1 (see Figure 2). Our working model is that T cell activation-induced phosphorylation of ZFP36 and CELF1 contributes to the up-regulation of transcripts encoding KSP components, and subsequent dephosphorylation of the RNA-BPs leads to down-regulation of these transcripts through ARE- or GRE-mediated mRNA decay. Thus, we suggest that ARE- and GRE-mediated mRNA decay plays a central role in the coordinate down-regulation of these genes following T cell activation through feedback inhibition of kinase signaling.

In this example, phosphorylation of key RNA-BPs, such as ZFP36 and CELF1, appears to coordinate the transient increased expression of multiple KSP components following T cell activation to allow T cell activation signals to be successfully transmitted. Thus, RNA-BP phosphorylation increases the expression of a network (or regulon) of transcripts encoding KSP components to further promote T cell activation. Later in the cellular activation program, ZFP36 and CELF1 become dephosphorylated, allowing the network transcripts encoding KSP components to undergo rapid mRNA decay. The downstream result would be to down-regulate components of KSP signaling to turn off cellular activation and bring the cell back to a state of quiescence.

**Figure 3:**
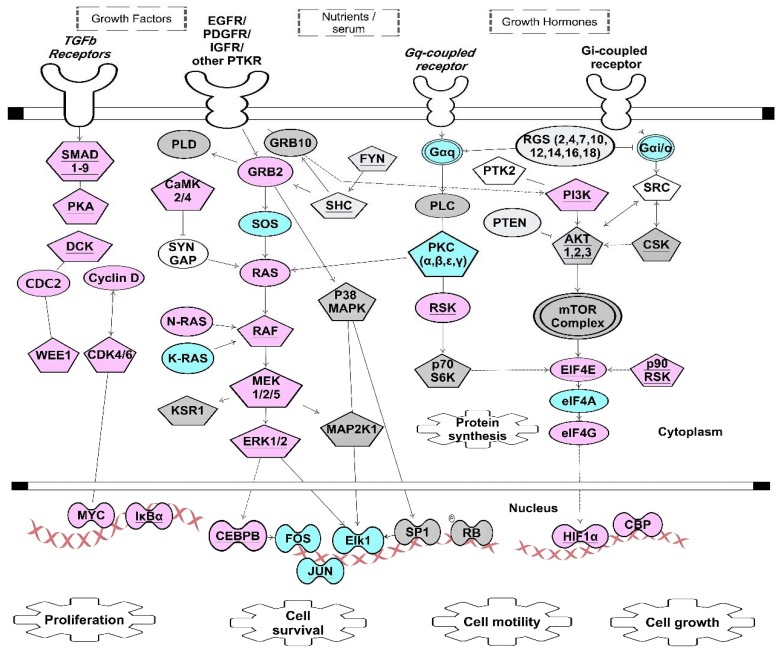
ARE- and GRE-signaling pathways stimulated through growth factors and growth hormones. This figure depicts simplified kinase signaling pathway downstream of growth factor and G protein-coupled receptors that are targets for CELF1, ZFP36 and ELAVL1 [31,61,104]. Proper expressions of these transcripts cooperatively contribute to the overall cellular signaling outcomes such as cell proliferation, cell survival, cell growth and cell motility. Pentagon-shape nodes represent kinase transcripts. Transcripts marked with underlined text were identified as CELF1 targets in resting T cells [61]. Transcripts in blue are targets of both CELF1 and ELAVL1 [31,61]. Transcripts in pink are targets of both ELAVL1 and ZFP36 [31,104]. Transcripts in grey are targets of ELAVL1 but not CELF1 or ZFP36 [31]. Underlined pink nodes represent transcripts that can be targets of all three proteins. Arrows indicate direct interactions and/or activations. Blunt-ended lines indicate inhibitory effects. This network diagram was built using Ingenuity Pathway Assistant Software.

We compared GRE-containing transcripts that were CELF1 targets [61] with ELAVL1 targets [31], and found numerous transcripts that were targets of both CELF1 and ELAVL1. RNA recognition sequences for CELF1 require precise GU repeats or overlapping GUUUG sequences, whereas the recognition sequence for ELAVL1 is less precise, and ELAVL1 binds to a variety of U-rich sequences, including AU-rich sequences, GU-rich sequences or a poly-U sequence. Many ARE-containing transcripts that are targets for ZFP36 are also targets for ELAVL1 [104]. Thus, ZFP36 and ELAVL1 appear to compete for a subset of ARE-containing target transcripts, and CELF1 and ELAVL1 compete for a subset of GRE-containing transcripts. Depending on which protein is more abundant and has higher affinity for the target binding site in a given target transcript, mRNA may undergo stabilization *versus* decay. We propose a model whereby phosphorylation of ZFP36 or CELF1 following activation of kinase signaling pathways, shifts the balance toward ELAVL1 binding to target transcripts, promoting transient stabilization of ARE- or GRE-containing mRNAs, including transcripts encoding KSP components. Figure 3 shows multiple examples of ARE- or GRE-containing transcripts that encode components of KSPs that are known targets of ZFP36, CELF1, and/or ELAVL1. The examples shown in Figure 3 suggest that feedback inhibition by AREs and GREs regulates KSPs in multiple settings, similar to what we have seen following T cell activation. Thus, it appears that phosphorylation of RNA-BPs through kinase signaling serves as a general mechanism to coordinately regulate the expression of networks of transcripts (RNA operons) which encode KSP components that control cell fate decisions, such as cell growth, proliferation, motility, or survival.

## 7. Conclusions

This review highlighted the role of kinase signaling pathways in the regulation of phosphorylation and function of RNA-binding proteins such as ZFP36, CELF1 and ELAVL1, that in turn function as posttranscriptional regulators of ARE- and GRE-containing mRNAs which encode components of KSPs. Such feedback inhibition mechanism is important for many cellular processes e.g. cell activation, limited proliferation and stress responses. A major priority for future research should be to design integrative studies to further elucidate the mechanisms by which AREs, GREs, RNA-BPs and also small regulatory RNAs coordinate signaling pathways involved in health and disease. For example, system level approaches should be applied to look at the interplay between differentially phosphorylated RNA-BPs and target transcripts to better understand functional outcomes of specific phosphorylation events. Immunoprecipitation of multiple RNA-BPs and identification of co-purified transcripts in single cell using high throughput sequencing technology would allow computational approaches to characterize a composite of regulatory regions within mRNAs and to provide information on how combinations of RNA-BPs function together. Proteomics studies will make possible identification of subcellular RNA-protein complexes, their interactions and trafficking. Animal models, to evaluate gain- or loss-of-function mutations on the functions of RNA-BPs, should be expanded to also assess the effect of phosphomimic or nonphosphorylatable mutations in RNA-BPs. These and similar genetic manipulations in mouse models should shed light on the functional relevance of feedback regulation of kinase signaling pathways by AREs and GREs. Finally, studies should be pursued to understand RNA-BP phosphorylation and downstream posttranscriptional networks in disease states, such as autoimmunity, immunodeficiency, and cancer.

## Abbreviations

ARE: AU-rich element
GRE: GU-rich element
RNA-BP: RNA-binding protein
UTR: 3’ untranslated region
KSP: kinase-signaling pathway
RRM: RNA-Recognition Motif
CHK2: cell cycle checkpoint kinase
PI3K: phosphatidylinositol 3-kinase
PKB: protein kinase B
PKC: Protein Kinase C
MEK1: mitogen-activated protein kinase kinase 1
MAPKs: mitogen-activated protein kinases
AMPK: AMP-activated kinase
CDK1: cyclin-dependent kinase 1
CHK2: cell cycle checkpoint kinase 2
DMPK: dystrophia myotonica protein kinase
TGFα/β: transforming growth factors-alpha and beta
GPCR: G protein-coupled receptor
